# Targeting the PRMT1 axis in cancer: from epigenetic plasticity to contextual therapeutic interventions

**DOI:** 10.3389/fonc.2026.1841266

**Published:** 2026-07-14

**Authors:** Shahper Nazeer Khan, Aamir Ahmad, Mohd Haris Siddiqui

**Affiliations:** 1 Integral Centre of Excellence for Interdisciplinary Research (ICEIR), Integral University, Lucknow, India; 2 Department of Biosciences, Integral University, Lucknow, India; 3 Translational Research Institute, Hamad Medical Corporation, Doha, Qatar; 4 Integral Institute of Agriculture Science and Technology, Integral University, Lucknow, India; 5 Department of Bioengineering, Integral University, Lucknow, India

**Keywords:** arginine methylation, epigenetic cross-talk, PRMT1 axis, PRMT1 inhibitors, therapeutic target

## Abstract

Protein arginine methyltransferase 1 (PRMT1), the predominant Type I arginine methyltransferase, is reported to play the key role in regulating cancer epigenetics. Its activity directs gene expression, chromatin structure and transcriptional activation by mediating asymmetric dimethylation of histone and non-histone proteins. Despite extensive characterization of PRMT1 as a dominant arginine methyltransferase, its paradoxical oncogenic and tumor-suppressive roles have remained conceptually fragmented. This review integrates isoform-specific biology, chromatin crosstalk, and emerging therapeutic strategies to resolve this paradox and provide a translational framework for PRMT1-targeted precision oncology. PRMT1 exhibits context-dependent activity. While it predominantly functions as an oncogenic regulator in both solid tumors and hematological malignancies, emerging evidence indicates that PRMT1 may also exert tumor-suppressive effects under specific metabolic, apoptotic, and microenvironmental contexts. This differential behavior is mediated by chromatin remodeling, transcription factor recruitment, enhancer-promoter interactions, and its interplay with key epigenetic regulators such as EZH2, leukemogenic fusion proteins like MLL-AF9, and non-coding RNAs. As a drug target, PRMT1 has gained significant attention, with several small-molecule inhibitors demonstrating efficacy in preclinical and early clinical studies. However, challenges such as off-target effects and resistance highlight the need for a deeper mechanistic understanding of its cancer-specific roles. By synthesizing current insights, this review elucidates PRMT1’s multifaceted role in cancer biology and underscores its potential as a target for precision oncology, laying the groundwork for future therapeutic strategies.

## Introduction

Protein arginine methylation is a vital post-translational modification catalyzed by the protein arginine methyltransferase (PRMT) family, which modulates the functional role of histone and non-histone proteins. This modification regulates different cellular processes, including transcription, RNA splicing, DNA damage repair, and signaling pathways. PRMTs are classified into three enzymatic subtypes as per their methylation pattern: Type I enzymes give asymmetric dimethylation of arginine residues; Type II enzyme activity results in symmetric dimethylation; and Type III enzymes induce monomethylarginine. Among these, PRMT1 is the predominant Type I methyltransferase, responsible for over 85% of asymmetric dimethylation activity in mammalian cells (1). PRMT1’s enzymatic activity influences chromatin structure and gene expression by methylating histones, such as histone H4 at arginine 3 (H4R3me2a), a mark associated with transcriptional activation. Beyond histones, PRMT1 modifies several non-histone substrates, including transcription factors (e.g., p53, NF- κB), RNA- binding proteins, and proteins involved in DNA repair, emphasizing its dynamic regulatory functions. These modifications are shown to be pivotal in maintaining cellular homeostasis, modulating processes such as cellular differentiation, proliferation, apoptosis, and the DNA damage response (2). Recent research highlights PRMT1 as a crucial player in cancer biology, with its dysregulation contributing to epigenetic reprogramming and cancer evolution. Overexpression of PRMT1 has been reported in a range of solid cancers, including breast, lung, colorectal, and pancreatic cancers, where it promotes oncogenic transcriptional pathways, malignant cell proliferation, and their metastatic potential (3). In hematological malignancies, the role of PRMT1 appears highly context-dependent. While numerous studies have demonstrated that PRMT1 promotes leukemogenesis through cooperation with MLL fusion proteins, HOXA9 signaling, and FLT3-ITD activation, emerging evidence suggests that PRMT1 may also participate in tumor-suppressive pathways under specific metabolic and apoptotic conditions. This duality in its functional regulation reflects its complex, context-dependent role of PRMT1 activity in cancer.

The major contribution by which PRMT1 influences cancer progression is its regulation of chromatin structure and transcription. PRMT1 mediated methylation of H4R3 promotes chromatin availability, easing transcriptional activation of oncogenes. Furthermore, PRMT1 cooperates with other chromatin modifiers, including the Polycomb group protein EZH2, and interacts with oncogenic fusion proteins such as MLL-AF9, which drive leukemogenesis by deregulating transcriptional balance critical for hematopoietic stem cell differentiation (4). Beyond its chromatin- related functions, PRMT1 modulates the activity of non-coding RNAs (ncRNAs), including microRNAs (miRNAs) and long non-coding RNAs (lncRNAs), thus impacting cancer- related signaling pathways (5). The therapeutic potential of targeting PRMT1 has gained significant attention recently, with small molecule inhibitors showing efficacy in preclinical and early clinical studies. Molecules like MS023 and GSK3368715 have demonstrated specific inhibition of PRMT1 activity, leading to suppressed cancer cell proliferation and enhanced susceptibility to chemotherapy and immune checkpoint blockers (6). Nevertheless, the idea of therapeutic targeting of PRMT1 still represent significant challenges, including its distinct functions in different regulatory pathways, unspecific activity, and further the emergence of resistance mechanisms. These limitations advocate the need for a better understanding of PRMT1’s various mechanistic roles in cancer biology. This review aims to give a comprehensive current understanding of PRMT1 in cancer epigenetics, emphasizing its dual role as an oncogene or tumor suppressor, its integration into the broader epigenetic crosstalk, and its potential translation therapeutic strategy for better clinical outcomes.

## PRMT1 as a regulator of cancer epigenetics

### Isoform-level complexity of PRMT1 in cancer

Isoforms-specific biology of PRMT1 is increasingly recognized as a critical determinant of its functional diversity in normal physiology and cancer, yet it remains under-reviewed compared to total PRMT1 expression. The PRMT1 gene undergoes complex alternative splicing to generate at least seven distinct protein isoforms (PRMT1-v1 through v7) with unique N-terminal sequences that influence enzymatic activity, substrate preference, and subcellular localization (7). Classic mechanistic studies demonstrated that these N-terminal differences change catalytic output and substrate selectivity, with isoforms such as v3 and v4 showing lower intrinsic activity and others exhibiting preferential methylation of distinct target sets. Isoform-specific interactome mapping confirmed divergent cellular roles: for instance, PRMT1-v1 is enriched in nuclear complexes regulating gene expression, whereas PRMT1-v2, which contains a functional nuclear export sequence and is predominantly cytoplasmic, associates with cytoskeletal regulators and promotes cancer cell survival and invasiveness in breast cancer models (8). Clinically, differential expression of PRMT1 splice variants correlates with tumor characteristics; PRMT1-v1 and PRMT1-v2 levels are elevated in breast tumors and linked to poor prognosis and epithelial-mesenchymal transition, while broader splicing complexity revealed by next-generation sequencing identified novel variant transcripts that could encode additional functional isoforms or regulatory noncoding RNAs (9, 10). Isoform diversity also intersects with key PRMT1 regulated processes such as alternative splicing and DNA damage responses: recent methylome profiling showed isoform-dependent regulation of splicing factor methylation, contributing to aberrant exon inclusion in breast tumors (10). Taken together, this body of evidence highlights that PRMT1’s cancer-relevant functions cannot be fully understood through total expression alone; dissecting isoform-specific expression, interactomes, and substrate networks is essential for predictive biomarker development, rational combination strategies, and potentially designing isoform-selective inhibitors or degrader approaches that improve therapeutic precision (**Table 1**).

**Table 1:** Isoform-level complexity of PRMT1 and its functional implications.

PRMT1 Isoform	Key structural feature (n-terminal differences)	Subcellular localization	Catalytic activity/substrate preference	Functional role/interactome insights	Clinical relevance
PRMT1-v1	Full-length, canonical sequence	Predominantly nuclear	High catalytic activity; strong H4R3 methylation	Interacts with chromatin regulators; controls transcriptional programs (9)	Elevated in breast tumors; associated with poor prognosis
PRMT1-v2	Contains NES (nuclear export sequence)	Mostly cytoplasmic	Methylates cytoplasmic proteins; altered substrate spectrum	Associates with cytoskeletal/signaling proteins; promotes survival/invasiveness (8)	Overexpressed in aggressive breast cancers; linked to EMT and metastasis
PRMT1-v3	Shorter N-terminal region	Mixed nuclear/cytoplasmic	Reduced intrinsic activity	Limited interactome; role unclear but may fine-tune methylation output (10)	Potential biomarker of low-activity PRMT1 states
PRMT1-v4	Alternative N-terminal truncation	Nuclear	Low catalytic activity; reduced H4R3me2a output	May regulate nuclear methylation buffering; interacts with fewer transcriptional complexes (1)	Could modulate response to PRMT1 inhibitors
PRMT1-v5	Distinct N-terminal splice pattern	Cytoplasmic	Moderate activity; possible RNA-binding protein preference	Emerging roles in RNA metabolism; requires further mapping (11)	May influence splicing phenotypes in tumors
PRMT1-v6	Rare transcript variant	Nuclear	Unknown; predicted weak catalytic activity	Under investigation; may encode regulatory functions	Low expression in tumors; biomarker potential unclear
PRMT1-v7	Highly diverged isoform; extended N-terminus	Nuclear	Potentially altered substrate binding	Could contribute to chromatin-specific methylation environments	Needs evaluation in clinical datasets
Novel isoforms (NGS-detected)	Dozens of transcript variants	Isoform-specific	Variable, uncharacterized	Some may function as non-coding RNAs or dominant-negative proteins (9, 10)	Represents an unexplored biomarker landscape and therapeutic opportunity

### Molecular functions and substrate landscape of PRMT1

**Figure 1:**
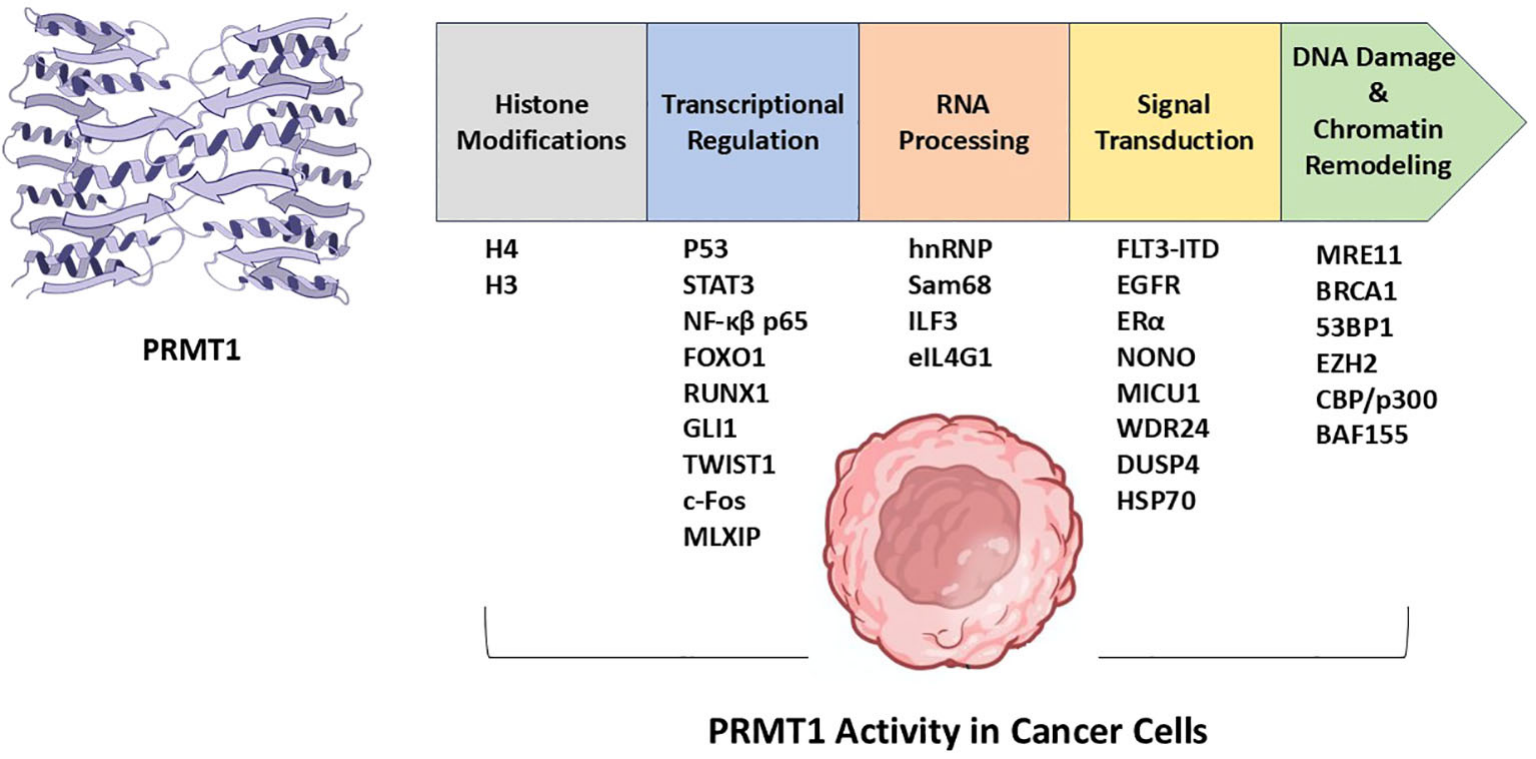
Multifunctional role of PRMT1 in cancer cells. PRMT1 regulates multiple oncogenic processes through arginine methylation of histone and non-histone substrates. PRMT1-mediated activity influences histone modification, transcriptional regulation, RNA processing, signal transduction, and DNA damage repair/chromatin remodeling. Through these pathways, PRMT1 promotes cancer cell proliferation, survival, metastasis, epigenetic reprogramming, and therapeutic resistance.

PRMT1 plays a vital part in modulating gene expression through its methyltransferase action, which targets both histones and non-histone proteins (**Figure 1**). Among its most studied post translational modification, PRMT1 catalyzes the asymmetric dimethylation of histone H4 at arginine 3 (H4R3me2a). This epigenetic mark is well established for promoting transcriptional activation by driving the recruitment of chromatin modifying complexes and transcriptional coactivators. These modifications increase open state of chromatin, resulting in active transcription of target genes. For example, in breast cancer cells, PRMT1 mediated H4R3me2a enhances the expression of oncogenes linked to tumor progression and metastasis (12). Beyond histone substrates, PRMT1 exhibit regulatory function by methylating crucial non-histone proteins, including transcription factors like p53 and NF- κB. This methylation affects their stability, subcellular localization, and DNA-binding activity. Moreover, PRMT1 impacts RNA- binding proteins and components of the splicing complex, thereby affecting alternate splicing patterns associated with oncogenic pathways (13). Recent research also emphasizes its role in enhancer-promoter interactions, forming chromatin structures that upregulate the transcription of oncogenic genes. The wide array of substrates targeted by PRMT1, including histones, transcription factors, RNA-binding proteins, and signaling molecules are summarized in **Table 2**.

**Table 2:** Key substrates of PRMT1 and their functions.

Category	Substrate	Residues	Function	References
Histone methylation	H4	R3me2a	Transcription activation	14
Transcription factors	STAT1	R31	Activates STAT1 transactivity	15
	C/EBPα	R35/156/165	Blocks the interaction with its corepressor, HDAC3	16
	RUNX1	R206/210	Interferes with binding to SIN3A	17
	FOXO1	R248/250	Stabilizes the FOXO1 protein	18
	MyoD	R121	Increases MyoD transactivity	19
	Nrf2	R437	Increases DNA-binding affinity and transactivity	20
	Twist1	R34	Facilitates repressive activity at the *E-cadherin* promoter	21
	p65/RelA	R30	Inhibits its own DNA-binding affinity	22
	GLI1	R597	Enhances the recruitment of GLI1 to target gene promoters	23
	BRCA1	Within the 504–802 region	Promotes BRCA1 recruitment to specific promoters	24
	c-Myc	R299, R346	Promotes c-Myc interaction with p300	25
	EZH2	R342	Prevents EZH2 target gene expression	26
	FOXP3	R48, R51	Enhances FOXP3 transcriptional activity	27
	PR	R637	Accelerates PR recycling and transcriptional activity	28
	RACO-1	R98, R109	Promotes c-Jun/AP1 activation	29
	RIP40	R240, R650, R948	Favors RIP140 nuclear export and prevents the recruitment of HDAC3	30
	TAF15	R203	Affects the subcellular localization of TAF-15 and enhances its transcriptional activity	31
	FUS/TLS	R216, R218, R242, R394	Participates in the nuclear cytoplasmic shuttling of FUS/TLS and enhances its transcriptional activity	32
mRNA splicing/alternative splicing	fibrillarin	–	Facilitates interaction with SMN	33
	GAR1	–	Facilitates interaction with SMN	33
	hnRNP A2	–	Regulates cytosolic/nucleus localization	34
	hnRNAP Q	–	Regulates cytosolic/nucleus localization	35
	hnRNP K	–	Promotes the interaction with c-Src	36
	RBM15	R578	Promotes ubiquitination by E3 ligase CNOT4	37
Translation	AVEN	–	Regulates translation in G-quadruplexes harboring mRNA	38
	TOP3B	R833/835	Localizes to stress granules	39
	rpS3	R64/65/67	Promotes ribosome assembly	40
Cell signaling	EGFR	R198/200	Increases binding affinity for EGF leading to dimerization of EGFR	41
	ASK1	R78/80	Promotes the association with thioredoxin	42
	Smad6	R74	Facilitates the dissociation of Smad6 from type I receptors	43
	Smad7	R57/67	Facilitates the dissociation of Smad7 from type I receptors	44
Cell cycle	CDK4	R55/73/82/163	Inhibits CDK-Cyclin D3 complex formation	45
	INCENP	R887	Facilitates interaction with AURKB	46
	UBAP2L	RGG/RG motif	Promotes alignment of chromosomes in metaphase	47
DNA damage response	MRE11	GAR motif	Activates exonuclease activity and recruits factors to damaged DNA	48, 49
	53BP1	GAR motif	Increases DNA-binding affinity	50, 51
	DNA polymerase β	R137	Interferes with binding with PCNA	52
	FEN1	R192	Interaction with PCNA and localization to damaged DNA foci	53

## Mechanistic basis of context-dependent PRMT1 function in cancer

### PRMT1 mediated transcriptional activation and chromatin remodeling

**Figure 2:**
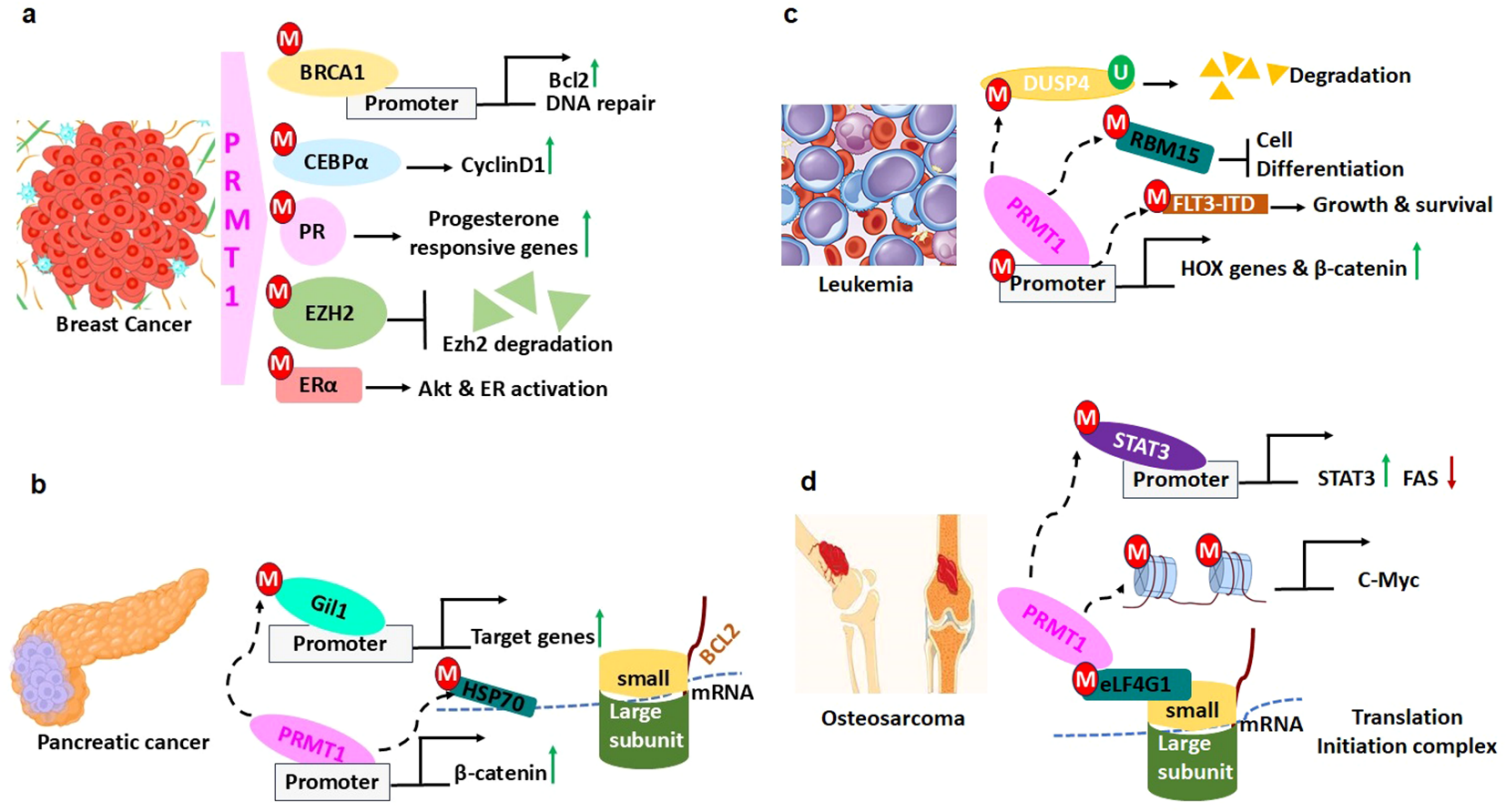
Cancer-promoting role of PRMT1-mediated arginine methylation in cellular dynamics. PRMT1 promotes oncogenic cellular dynamics through arginine methylation of histone and non-histone substrates involved in transcriptional regulation, signal transduction, RNA processing, and chromatin remodeling. In breast cancer, PRMT1 enhances ERα signaling, Cyclin D1 expression, BRCA1-mediated DNA repair, and EZH2 stabilization. In pancreatic cancer, PRMT1 activates Gli1 and β-catenin signaling and regulates HSP70-associated survival pathways. In leukemia, PRMT1 promotes FLT3-ITD signaling, RBM15-associated leukemogenic programs, and HOX/β-catenin-driven transcription. In osteosarcoma, PRMT1 regulates STAT3 signaling, c-Myc expression, and eIF4G1-mediated translational initiation. Collectively, these mechanisms contribute to cancer cell proliferation, survival, metastasis, and therapeutic resistance.

PRMT1 functions as a major epigenetic activator in multiple cancers through asymmetric dimethylation of histone and non-histone substrates that regulate chromatin accessibility, enhancer activation, and oncogenic transcriptional programs. In breast cancer, PRMT1 promotes tumor progression through H4R3me2a-mediated transcriptional activation of EMT-associated genes, particularly *ZEB1*, thereby facilitating epithelial-to-mesenchymal transition (EMT) and metastatic dissemination (54). In breast cancer, p300/CBP acts as a transcriptional coactivator that enhances chromatin accessibility and oncogenic gene expression associated with proliferation, EMT, and therapeutic resistance. Increased p300/CBP activity has also been associated with aggressive disease progression, highlighting the importance of epigenetic coactivator networks in PRMT1-mediated transcriptional regulation. PRMT1 further enhances oncogenic chromatin remodeling by stabilizing EZH2 through arginine methylation, which potentiates Polycomb Repressive Complex 2 (PRC2) activity and suppresses tumor suppressor genes such as *P16* and *P21* (26). Similarly, in colorectal cancer, PRMT1-mediated methylation of histone H4R3 and EGFR enhances receptor signaling and downstream transcriptional activation of oncogenic pathways, thereby supporting tumor growth and metastasis (55). PRMT1 additionally methylates NONO, contributing to colorectal cancer progression independent of KRAS mutational status (56). In gastric cancer, PRMT1 activates β-catenin signaling and stabilizes c-Fos to enhance AP-1 transcriptional activity, collectively promoting tumor growth and transcriptional dysregulation (57). In pancreatic cancer, PRMT1 amplifies transcriptional outputs of β-catenin and Gli1 through arginine methylation, thereby driving oncogenic signaling and metastatic progression (23). Collectively, these studies demonstrate that PRMT1-mediated chromatin remodeling and enhancer-associated transcriptional activation constitute major mechanisms underlying its oncogenic activity across solid tumors (**Figure 2**).

### PRMT1 regulation of EMT, invasion and metastatic plasticity

PRMT1 critically regulates EMT and metastatic plasticity through methylation-dependent modulation of transcription factors and signaling pathways associated with cellular migration and invasiveness. In non-small cell lung cancer (NSCLC), PRMT1 methylates Twist1, repressing E-cadherin expression and promoting metastatic dissemination (58). Likewise, PRMT1 induces EMT in hepatocellular carcinoma (HCC) through activation of TGF-β1/Smad signaling pathways, thereby increasing invasiveness and metastatic potential (59). In ovarian cancer, PRMT1-mediated methylation of FAM98A enhances proliferation, migration, and invasion (60). Similarly, in esophageal cancer, PRMT1 stabilizes Gli1 through arginine methylation, accelerating Hedgehog signaling and tumor progression (61, 62). These findings collectively indicate that PRMT1 acts as a central regulator of metastatic transcriptional programs and EMT-associated cellular plasticity in diverse tumor contexts.

### PRMT1 in DNA damage repair, therapeutic resistance, and cancer cell survival

A major oncogenic function of PRMT1 involves regulation of DNA damage repair and adaptive resistance to anticancer therapies. In breast cancer, PRMT1 methylates BRCA1 to enhance DNA repair efficiency and resistance to radiotherapy and PARP inhibition, while simultaneously promoting anti-apoptotic signaling through induction of BCL2 expression (63). In NSCLC, PRMT1 enhances chemoresistance through methylation of SOX2, thereby increasing cancer stemness, and through FEN1 methylation, which facilitates DNA repair machinery activity (64, 65). In pancreatic cancer, PRMT1-mediated methylation of HSP70 stabilizes anti-apoptotic proteins including BCL2, contributing to survival under therapeutic stress (66). Likewise, in colorectal cancer, PRMT1-mediated EGFR activation contributes to resistance against EGFR-targeted therapies (56). These observations collectively suggest that PRMT1 promotes therapy adaptation through coordinated regulation of DNA repair, apoptosis resistance and stemness-associated signaling pathways.

### PRMT1 in metabolic reprogramming and oncogenic signaling

PRMT1 additionally contributes to tumor progression through modulation of metabolic and signaling networks that sustain cancer cell proliferation. In lung cancer, PRMT1 regulates mitochondrial calcium uptake and oxidative phosphorylation, thereby supporting metabolic fitness and proliferative potential (58). In hepatocellular carcinoma, PRMT1 enhances STAT3 signaling through arginine methylation while indirectly facilitating phosphorylation-dependent STAT3 activation and downstream transcriptional signaling (67). PRMT1 also interacts with the GATOR2 complex to inhibit GATOR1 activity and deregulate mTORC1 signaling, promoting HCC proliferation (68). These findings position PRMT1 as an important integrator of epigenetic and metabolic oncogenic signaling pathways.

### PRMT1 in leukemogenesis and stem cell maintenance

PRMT1 plays a critical role in hematologic malignancies by sustaining leukemic stemness and reinforcing oncogenic transcriptional programs. In leukemia, PRMT1 cooperates with HOXA9, β-catenin, and MLL fusion proteins to promote recruitment of transcriptional complexes associated with H4R3 asymmetric dimethylation and leukemic self-renewal (4). PRMT1 also methylates FLT3-ITD, amplifying oncogenic signaling and contributing to leukemogenesis and chemoresistance (69). These observations indicate that PRMT1 functions as a key epigenetic dependency in leukemia through maintenance of stem-like transcriptional states and oncogenic signaling circuitry. Although PRMT1 predominantly exhibits oncogenic functions in most cancers, including AML and other hematological malignancies, several studies have reported anti-proliferative and pro-apoptotic activities under specific cellular contexts. Collectively, current evidence indicates that PRMT1 primarily functions as an oncogenic driver in AML and related leukemias; however, isolated studies have reported anti-proliferative effects mediated through mitochondrial stress and apoptotic pathways, highlighting the context-dependent nature of PRMT1 biology.

### Context-dependent tumor-suppressive functions of PRMT1

**Figure 3:**
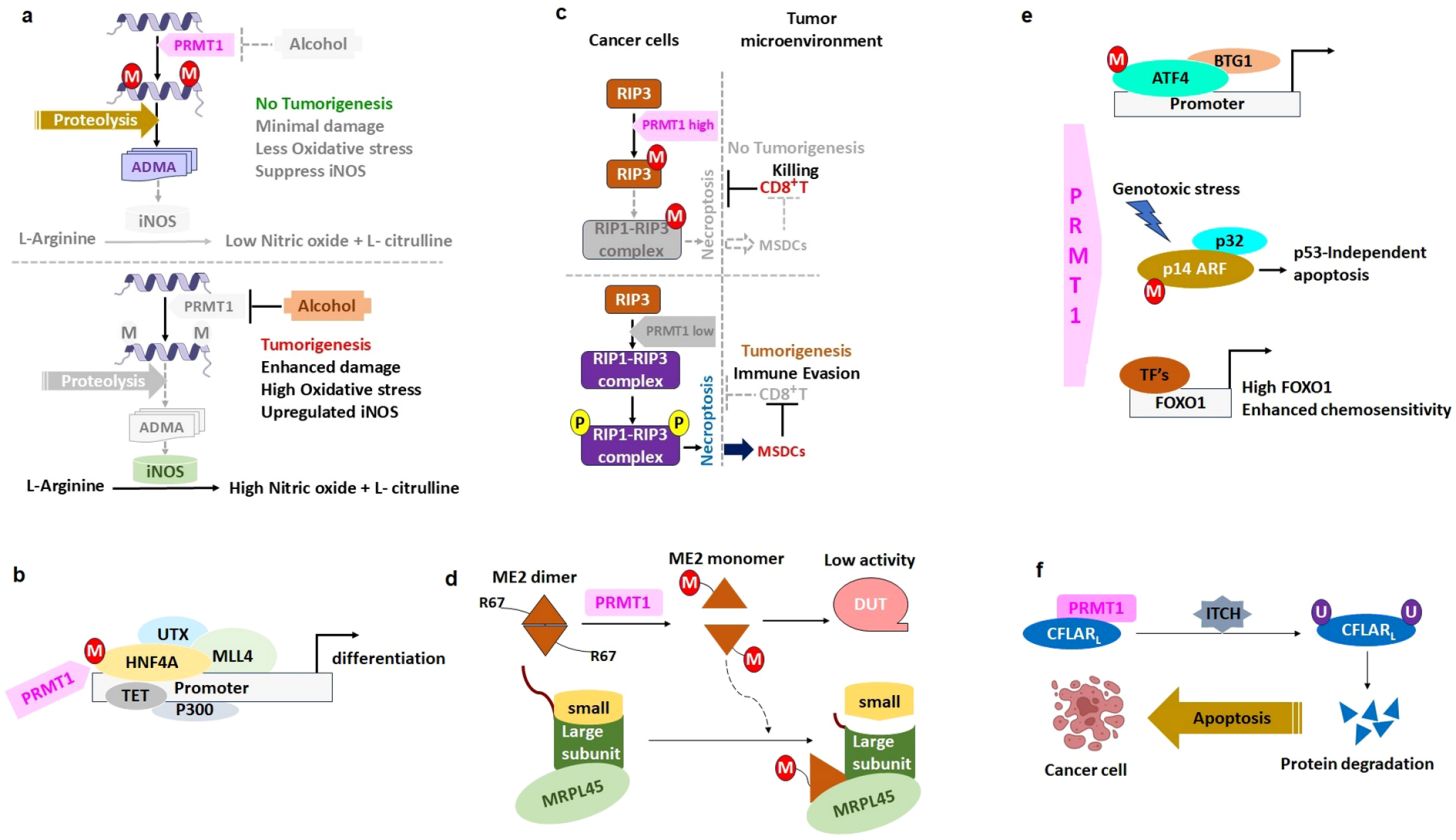
Tumor-suppressive mechanisms of PRMT1-mediated arginine methylation in cancer. **(a)** PRMT1-mediated ADMA production suppresses iNOS activity and oxidative stress, thereby reducing alcohol-associated liver tumorigenesis. **(b)** PRMT1 promotes HNF4A-associated differentiation signaling through epigenetic transcriptional regulation. **(c)** PRMT1-mediated methylation of RIP3 inhibits necroptosis-associated immune evasion and tumor progression. **(d)** PRMT1 methylation of ME2 disrupts mitochondrial ribosome assembly and impairs leukemic cell proliferation. **(e)** PRMT1 promotes tumor suppression through activation of ATF4-dependent apoptosis, p14ARF-mediated p53-independent apoptosis, and FOXO1-associated chemosensitivity pathways. **(f)** PRMT1 induces CFLAR degradation through ITCH-mediated ubiquitination, thereby promoting apoptosis in cancer cells. Collectively, these mechanisms highlight the context-dependent tumor-suppressive functions of PRMT1 in cancer.

Although PRMT1 predominantly exhibits oncogenic activity in most cancers, including AML and other hematological malignancies, several studies have reported tumor-suppressive functions in specific metabolic, apoptotic, and tissue-dependent contexts. In alcohol-associated liver cancer, inhibition of PRMT1-mediated methylation at the HNF4A promoter reduces HNF4A expression, thereby aggravating oxidative stress, inflammation, and Wnt/β-catenin activation to promote hepatocarcinogenesis (70). PRMT1-mediated asymmetric dimethylarginine (ADMA) formation additionally suppresses inducible nitric oxide synthase activity, thereby limiting reactive nitrogen species generation and inflammatory liver injury (71). In necrotic colon cancer, PRMT1 methylates RIP3 and suppresses RIP3 phosphorylation, thereby inhibiting necroptosis-associated immune evasion and tumor progression (72). PRMT1 also exerts tumor-suppressive effects through regulation of mitochondrial stress and apoptosis. In leukemia, PRMT1-mediated methylation of ME2 disrupts mitochondrial ribosome assembly and impairs mtDNA synthesis, thereby suppressing leukemia cell proliferation (73). As a result, the progression of leukemia is significantly regress. PRMT1 additionally activates ATF4 through BTG1-dependent methylation, promoting transcription of pro-apoptotic genes (74). In NSCLC, PRMT1 promotes ubiquitination and degradation of the anti-apoptotic protein CFLARL through ITCH-mediated mechanisms thereby increasing the apoptosis of NSCLC cells (75). Similarly, in pancreatic cancer, PRMT1-mediated asymmetric dimethylation of arginine residues within the NLS/NoLS region of p14ARF, specifically at R87, R88, R96, and R99, disrupted its interaction with nucleophosmin (NPM). This modification led to the redistribution of both p14ARF and NPM from the nucleolus to the nucleus and cytoplasm, thereby triggering p53-independent apoptotic pathways and contributing to improved patient outcomes. (76). Notably, PRMT1 is also been reported to exhibit context-dependent dual function in gastric cancer, as it has been reported to both facilitate epithelial–mesenchymal transition (EMT) and suppress tumor cell proliferation. Furthermore, reduced PRMT1 expression impairs the nuclear accumulation of FOXO1 and diminishes responsiveness to chemotherapeutic agents, thereby increasing the risk of post-adjuvant chemotherapy relapse and contributing to poor clinical outcomes. (77, 78). Taken together, these observations collectively demonstrate that PRMT1 can also exert context-dependent tumor suppressive functions through regulation of oxidative stress, mitochondrial dysfunction, necroptosis, and apoptosis signaling pathways in specific cancer settings (**Figure 3**). These findings further indicates that PRMT1 context-dependent function in cancer is governed by multiple determinants, including tumor lineage, substrate availability, chromatin accessibility, interacting epigenetic regulators metabolic state and oncogenic mutational background. Furthermore, isoform-specific localization and signaling interactions may further influence whether PRMT1 exhibits oncogenic or tumor-suppressive effects within distinct cellular environments.

## Crosstalk of PRMT1 with other epigenetic regulators

### PRMT1 and histone variation

PRMT1 marked asymmetric dimethylation, particularly at histone H4 on arginine 3 (H4R3me2a), is a foundation of transcriptional activation. This modification infrequently functions as standalone; rather, it interplays with other histone marks like acetylation, phosphorylation, and ubiquitination, integrating into a complex network to regulate chromatin availability and transcriptional dynamics. H4R3me2a often acts in coordination with acetylation marks, such as H3K27ac and H3K9ac, to sustain transcriptionally active chromatin state. For instance, in breast cancer, H4R3me2a facilitates the recruitment of histone acetyltransferases like CBP/p300, increasing histone acetylation in proximity and resulting in higher expression of oncogenes involved in cell proliferation and metastatic progression (79). This induces enhancer- promoter loops essential for maintaining oncogenic transcriptional activity. PRMT1 also plays a critical part in the DNA damage response (DDR) in coordination with histone phosphorylation. When histone H2AX is phosphorylated (γH2AX) at a specific site on DNA double-strand, PRMT1 exhibits methylation at H4R3. This methylation enhances the recruitment of repair proteins, like MDC1, thereby triggering DDR signaling (80). Impairment in this crosstalk may lead to genomic instability, a hallmark of numerous cancers. Additionally, PRMT1 influences ubiquitination patterns by affecting substrate availability and recognition. In glioblastoma, H4R3me2a counters the repressive ubiquitination of H2A at lysine 119 (H2AK119ub), catalyzed by Polycomb repressive complex 1 (PRC1). This shift destabilizes repressive chromatin state and ensures transcriptional activation (81). By regulating chromatin structure, PRMT1 drives transitions from compact heterochromatin to open state chromatin structures, thereby enabling transcription factor binding and RNA polymerase II activity. In leukemia, PRMT1 in synergy with SWI/SNF chromatin remodelers destabilize repressive chromatin, promoting the activation of transcriptional activity essential for leukemic stem cell renewal (55).

### Interaction with fusion proteins (e.g., MLL- AF9)

In hematological malignancies, PRMT1 interaction with oncogenic fusion proteins illustrates its mechanistic role in epigenetic dysregulation. In acute myeloid leukemia (AML), PRMT1 is recruited by the fusion protein MLL- AF9 to specific gene loci, where it methylates H4R3. This modification further enhances H3K79 methylation by DOT1L, thereby upregulating leukemogenic genes like HOXA9 and MEIS1 (4). Concurrently, PRMT1 methylation antagonizes the repressive marks such as PCR2 mediated H3K27, thereby destabilizing the repressive chromatin structures. This interplay creates self-regulatory loop that drive self-renewal of leukemic stem cell and inhibit differentiation. Combination therapies targeting PRMT1 alongside DOT1L or BET inhibitors have shown potential in preclinical models for reversing these transcriptional aberrations (82).

### Regulation by non-coding RNAs

Non-coding RNAs (ncRNAs) add another layer of regulation to PRMT1 activity. Long non-coding RNAs (lncRNAs) like NEAT1 and HOTAIR interact with PRMT1 to modulate its localization and function. In prostate cancer, NEAT1 recruits PRMT1 to AR-dependent enhancers resulting in the transcription of genes involved in cancer growth and survival (14). HOTAIR stabilizes the EZH2-PRMT1 complex, promoting coordinated deposition of activating and repressive histone marks at specific loci, which contributes to oncogenic transcriptional reprogramming (83, 84). MicroRNAs (miRNAs) predominantly act as negative regulators of PRMT1 by binding to its mRNA and suppressing its expression, as demonstrated by miR-503, miR-494-3p and miR-455-5p, in various cancers and diseases (85–87). Direct positive regulation of PRMT1 by miRNAs is not well-documented, likely because miRNAs generally function to repress target gene expression. However, miRNAs may indirectly influence PRMT1 levels by targeting upstream repressors or modulating signaling pathways such as the mTOR-STAT1 axis that regulate PRMT1 expression (88). Additionally, PRMT1 itself plays a crucial role in miRNA biogenesis by methylating components of the microprocessor complex, thereby facilitating the maturation of several key miRNAs, and acts as a transcriptional coactivator promoting the expression of specific pri-miRNAs (89). This intricate reciprocal relationship between miRNAs and PRMT1 highlights the complexity of epigenetic and post-transcriptional regulation involved in health and disease. RNA-binding proteins (RBPs) also impact PRMT1 action by stabilizing its mRNA or easing its recruitment to chromatin. HuR, an RBP overexpressed in pancreatic cancer, stabilizes PRMT1 transcripts, enhancing aberrant arginine methylation patterns and supporting cancer progression (13). Proteins such as FUS and hnRNPK further directs PRMT1 to specific loci, linking histone methylation with RNA splicing and transcriptional regulation.

### Interplay with key epigenetic players

PRMT1 operates within a densely interconnected epigenetic landscape, and growing evidence indicates extensive crosstalk between PRMT1 and other chromatin-modifying enzymes that shapes transcriptional output and therapeutic vulnerabilities. PRMT1 mediated H4R3me2a deposition promotes enhancer activation by facilitating H3K27 acetylation and stabilizing BRD4 binding at super-enhancers, thereby amplifying MYC-driven transcriptional programs; accordingly, PRMT1 inhibition markedly enhances the efficacy of BET inhibitors in AML, neuroblastoma, and triple-negative breast cancer models (90, 91). PRMT1 also intersects with DNA methylation machinery, as its loss increases DNMT1 occupancy and promoter hypermethylation, whereas dual PRMT1–DNMT1 blockade restores expression of differentiation genes and improves responses in myeloid malignancies (11, 92). Functional cooperation between PRMT1 and histone acetylation has also been demonstrated, with HDAC inhibition potentiating transcriptional collapse upon PRMT1 loss in lymphoma and breast cancer (93). Conversely, PRMT1 antagonizes Polycomb repression, and PRMT1 depletion enhances PRC2 recruitment and H3K27me3 accumulation; therefore, combining PRMT1 and EZH2 inhibitors prevents compensatory silencing and produces synthetic lethality in breast and prostate cancer (94). Finally, PRMT1 exhibits compensatory redundancy with PRMT5, and reciprocal upregulation of PRMT5 upon PRMT1 inhibition (and vice versa) represents a key resistance mechanism. Dual inhibition of PRMT1 and PRMT5 disrupts both ADMA and SDMA methylation pathways, leading to catastrophic splicing defects and potent anti-leukemic activity in MYC-driven and MLL-rearranged leukemias (63, 95). Collectively, these findings position PRMT1 as a central epigenetic hub whose interactions with BET, DNMT, HDAC, EZH2, and PRMT5 pathways create rich opportunities for rational combination therapies as suggested in **Table 3**.

**Table 3:** Crosstalk of PRMT1 with major epigenetic modifiers and therapeutic implications.

Epigenetic drug class	Mechanistic basis of crosstalk with PRMT1	Biological consequences	Therapeutic implications	Key supporting studies (2021–2024)
BET inhibitors (BRD)	PRMT1-mediated H4R3me2a increases H3K27ac, promoting BRD4 recruitment to enhancers. PRMT1 inhibition reduces BRD4 enhancer binding.	Reduced MYC transcription, impaired super-enhancer activity, decreased oncogenic transcriptional programs.	Strong synergy of PRMT1i + BETi in MYC-driven cancers and AML; prevents enhancer reactivation resistance.	37, 57, 90, 91, 96
DNMT inhibitors	PRMT1 regulates DNMT1 chromatin recruitment; PRMT1 loss increases promoter DNA methylation in some loci.	Altered DNA methylation landscapes; partial reactivation of differentiation genes with combined inhibition.	PRMT1i + DNMTi enhances epigenetic reprogramming in AML; deeper differentiation responses.	11, 92, 97
HDAC inhibitors	PRMT1 cooperates with histone acetylation to activate transcription; PRMT1i destabilizes oncogenic transcriptional complexes.	Increased chromatin accessibility; synthetic lethality in cancers dependent on hyperactive transcription.	Combination benefits in lymphoid cancers and tumors with enhancer overactivation.	12, 93
EZH2 inhibitors (PRC2)	PRMT1 blocks PRC2 recruitment; PRMT1i increases H3K27me3 and EZH2 occupancy. EZH2i reverses PRC2 repression in PRMT1-high tumors.	Compensation between activation (PRMT1) and repression (EZH2) pathways; bidirectional chromatin plasticity.	PRMT1i + EZH2i synergizes in AML, breast cancer, prostate cancer.	26, 66, 84
PRMT inhibitors	PRMT1 (ADMA) and PRMT5 (SDMA) exhibit functional redundancy; inhibition of one upregulates the other.	Resistance to single-agent PRMT inhibition; collapse of methylation buffering when both are inhibited.	Dual PRMT1i + PRMT5i causes synthetic lethality in MYC-high and MLL-r leukemias.	26, 98–101

## PRMT1 in the tumor microenvironment

The tumor microenvironment (TME) represents a dynamic network among cancer cells and its proximal environment, including immune cells, stromal cells, extracellular matrix (ECM), and cytokines. Recent studies have illuminated the vital role of PRMT1 within the TME, affecting cancer progression, immune evasion, and drug resistance (2, 13).

### PRMT1 in innate immune and inflammatory regulation

In macrophages, PRMT1-mediated asymmetric dimethylation of NF-κB regulates its transcriptional activity and promotes expression of pro-inflammatory cytokines including IL-6, IL-1β, and TNF-α (79, 102). These cytokines contribute to tumor progression and metastasis. PRMT1 has also been associated with polarization toward the tumor-supportive M2 macrophage phenotype, which promotes immunosuppression and tissue remodeling. In natural killer (NK) cells, PRMT1 suppresses expression of activating receptors such as NKG2D through methylation-dependent transcriptional repression, thereby reducing NK cell-mediated cytotoxicity against tumor cells (103). Furthermore, PRMT1 negatively regulates type I interferon signaling through methylation of IRF7, weakening innate antitumor immune responses.

### PRMT1 in adaptive immunity and antigen presentation

PRMT1 critically regulates adaptive immune responses through modulation of T-cell differentiation, immunosuppressive signaling, and antigen presentation pathways. PRMT1-mediated methylation of STAT3 enhances its stability and transcriptional activity, promoting differentiation of naïve CD4+ T cells into Th17 cells (104). PRMT1 has additionally been linked to regulatory T-cell (Treg) expansion through epigenetic regulation of FOXP3, thereby contributing to immunosuppressive remodeling of the TME. In myeloid-derived suppressor cells (MDSCs), PRMT1-mediated methylation of STAT1 and IRF1 enhances PD-L1 expression, suppressing T-cell activation and facilitating tumor immune escape (88). Notably, STAT1 exhibits context-dependent roles in cancer. While classically associated with interferon-mediated tumor suppressive responses and immune surveillance, persistent STAT1 activation in certain tumors has also been linked to chronic inflammatory signaling, immune evasion, and therapeutic resistance. PRMT1 also regulates antigen presentation by suppressing transcription of MHC class II molecules in dendritic cells, thereby reducing dendritic cell-mediated T-cell activation (105). Furthermore, PRMT1 suppresses MHC class I expression in tumor and myeloid cells, limiting CD8+ cytotoxic T lymphocyte priming and infiltration within the TME (106). Together, these findings establish PRMT1 as a key regulator of adaptive immune suppression and tumor immune evasion.

### PRMT1 in stromal remodeling and therapeutic targeting within the TME

Beyond immune regulation, PRMT1 contributes to stromal remodeling and extracellular matrix (ECM) organization within the TME. In cancer-associated fibroblasts (CAFs), PRMT1 regulates expression of chemokines such as CXCL12, which recruit Tregs and MDSCs to tumor sites and enhance local immunosuppression (107). PRMT1 additionally modulates ECM remodeling through regulation of matrix metalloproteinases (MMPs), contributing to formation of physical barriers that restrict immune-cell infiltration and facilitate immune escape. In hepatocellular carcinoma (HCC), PRMT1-mediated methylation of malic enzyme 2 (ME2) promotes tumor growth and migration, linking PRMT1 activity to metabolic adaptation within the TME (108). Given its multifaceted immunomodulatory role, PRMT1 represents a promising therapeutic target for reprogramming the TME. PRMT1 inhibition has been shown to reduce PD-L1 expression, restore T-cell activation, and enhance antitumor immunity (107, 109). Combination therapies involving PRMT1 inhibitors and immune checkpoint blockade, including anti-PD-1 and anti-CTLA-4 therapies, have demonstrated enhanced antitumor responses and improved T-cell cytotoxic activity in preclinical models (110).

## Therapeutic targeting of PRMT1: mechanistic and translational perspectives

The central role of PRMT1 in oncogenic transcriptional regulation, epigenetic remodeling, metabolic adaptation, and therapeutic resistance has established it as an attractive therapeutic target in cancer. Recent advances in medicinal chemistry and epigenetic drug discovery have enabled the development of multiple PRMT1-targeting compounds with promising preclinical and early clinical activity. However, their clinical translation remains limited by challenges including target selectivity, compensatory resistance mechanisms, toxicity, and the context-dependent dual functions of PRMT1 in cancer biology.

### Pan-type I PRMT inhibitors

Recent advances in medicinal chemistry have enabled the development of pan-Type I PRMT inhibitors that broadly suppress asymmetric arginine methylation mediated by multiple PRMT family members, including PRMT1, PRMT3, PRMT4, PRMT6, and PRMT8. Among these, MS023 is one of the most extensively characterized compounds. Mechanistically, MS023 inhibits Type I PRMT-mediated methylation, including PRMT1-dependent H4R3me2a deposition associated with enhancer activation and oncogenic transcriptional regulation (100, 111). Preclinical studies demonstrated that MS023 reduces global H4R3me2a levels and suppresses proliferation of leukemia and carcinoma cell lines dependent on PRMT1 signaling. In breast cancer models, MS023-mediated inhibition of enhancer-associated methylation disrupted oncogenic transcriptional activation and impaired tumor growth (99). Although pan-Type I inhibitors exhibit broad antitumor activity, their reduced selectivity may also contribute to unintended epigenetic reprogramming and toxicity due to simultaneous inhibition of multiple PRMT family proteins. For example, concomitant disruption of PRMT5-associated methylation pathways may alter RNA splicing and transcriptional homeostasis, potentially aggravating off-target effects (93). Furthermore, adaptive resistance mechanisms including compensatory upregulation of alternative methyltransferases and alterations in SAM biosynthesis may limit therapeutic efficacy. These challenges highlight the need for next-generation inhibitors with improved selectivity and reduced systemic toxicity. Structurally, PRMT inhibitors target the conserved S-adenosyl methionine (SAM)-binding catalytic pocket, and crystallographic studies have provided important insights into optimizing specificity while minimizing off-target inhibition (110). A comparative overview of PRMT inhibitors, including IC50 values, selectivity profiles, and clinical status, is summarized in **Table 4**.

**Table 4:** PRMT1 inhibitors.

Categorization	Name	Mechanism of action	PRMT1 IC_50 (nM)_	References
Selective	CTS2190*	Active site of PRMT1	0.315	112
	C7280948	Active site of PRMT1	0.0128	113
	TC-E 5003	Active site of PRMT1	1.50	114
	ZJG51	Active site of PRMT1	0.0118	115
	ZJG58	Active site of PRMT1	0.0071	115
Preferential selective	AML1	Substrate competitive	0.0088	116
Non-selective	MS023	Substrate competitive	0.030	117
	GSK3368715*	Substrate competitive	0.031	98
	II757	SAM competitive	0.0164	118

*Clinical trials.

### Selective PRMT1 inhibitors

Selective PRMT1 inhibitors have emerged as a promising strategy to specifically suppress PRMT1-dependent oncogenic signaling while minimizing broader epigenetic disruption associated with pan-Type I inhibition. GSK3368715 is among the most clinically advanced PRMT1-preferential inhibitors and demonstrates improved pharmacokinetic properties with significant antitumor activity in multiple malignancies. Preclinical studies revealed that GSK3368715 suppresses oncogenic transcriptional programs and induces apoptosis in cancers exhibiting elevated PRMT1 expression, including pancreatic and lung cancers (119). In multiple myeloma models, GSK3368715 additionally reduced chemoresistance through suppression of DNA repair-associated pathways, supporting its potential utility in combination therapies (120). Therapeutic activity has also been reported in pulmonary disorders (121) and solid tumors (122). Despite these advances, selective PRMT1 inhibition remains challenged by the highly conserved catalytic domains shared among PRMT family members. Achieving isoform specificity while preserving physiological PRMT1 functions involved in hematopoiesis, immune regulation, differentiation, and DNA repair remains an important translational consideration. Consequently, therapeutic strategies involving partial inhibition, context-dependent targeting, and biomarker-guided patient selection may improve therapeutic windows while minimizing hematologic and immunologic toxicities. Emerging drug development approaches are therefore increasingly focused on exploiting subtle structural differences within PRMT1 catalytic and substrate recognition domains to achieve greater isoform selectivity and reduced off-target activity.

### Disease-specific therapeutic applications

The integration of PRMT1 inhibition into precision oncology strategies has generated significant interest in disease-specific therapeutic applications and rational combination therapies. In breast and carcinoma models, PRMT1 inhibition combined with anti-PD-1 immune checkpoint blockade enhanced T-cell infiltration and prolonged tumor suppression, suggesting that PRMT1 targeting may improve tumor immunogenicity and augment antitumor immune responses (123). Similarly, PRMT1 inhibitors have been proposed to synergize with immune checkpoint inhibitors through modulation of cancer-associated immune signaling pathways (82). However, most evidence supporting PRMT1i–ICI combinations remain preclinical and requires prospective clinical validation. Strong mechanistic rationale additionally supports combining PRMT1 inhibitors with DNA damage repair-targeted therapies such as PARP inhibitors. PRMT1 inhibition suppresses DNA repair signaling and sensitizes cancer cells to genotoxic stress, thereby enhancing susceptibility to PARP inhibition (124). Tumors harboring DNA repair deficiencies may therefore represent particularly suitable candidates for PRMT1-based synthetic lethality strategies. Furthermore, biomarker-driven patient stratification approaches have identified elevated PRMT1 expression, MYC amplification, TP53 mutations, and PRMT1-dependent transcriptional signatures as potential predictive markers of therapeutic responsiveness (5). Transcriptomic and proteomic profiling may further identify patient subsets likely to benefit from PRMT1 inhibitor combinations involving PARPi, BETi, HDACi, EZH2i, or immune checkpoint blockade (5, 98) (**Table 5**). Nevertheless, the translational application of PRMT1-targeted therapies remains limited by incomplete biomarker validation, compensatory resistance mechanisms, and the context-dependent dual functions of PRMT1 in cancer biology. Therefore, future clinical development will require integrated biomarker-guided strategies, rigorous pharmacodynamic monitoring, and careful evaluation of toxicity and therapeutic selectivity to optimize patient outcomes.

**Table 5:** Clinical translation & biomarker roadmap for PRMT1 inhibitors.

Step	Goal	Sample/assay	Key biomarkers/readouts	Clinical/therapeutic implications
Preclinical Validation	Confirm PRMT1 dependency, define PD & predictive biomarkers	*In Vitro* cancer models: cell lines, organoids & PDX	PRMT1 mRNA/protein, H4R3me2a, PRMT1 isoforms, transcriptional signatures, splicing stress	Identify responsive tumors, guide dose selection, nominate combinations (BETi, DNMTi, PARPi, PRMT5i)
Early Clinical Development (Phase 1/Window-of-Opportunity)	Demonstrate target engagement, establish safety, identify biomarker-positive cohorts	Tumor biopsy (FFPE + frozen), plasma, PBMCs	H4R3me2a reduction (validated PD marker), substrate methylation assays, γ-H2AX, exploratory ctDNA dynamics, RNA-seq signatures	Select patients for expansion cohorts, measure early PD response, de-risk toxicity
Biomarker Assay Validation	Develop CLIA-grade assays, define cutpoints	IHC, LC-MS/MS, RNA signature panels, digital pathology	PRMT1, H4R3me2a, substrate methylation, RNA dependency signatures, ctDNA	Enable biomarker-guided patient stratification; companion diagnostics remain investigational
Rational Combination Strategy	Prioritize synergistic combinations	Preclinical screens, genomic/epigenomic profiling	Pathway activation (MYC, BRD4, DDR, splicing)	Guide selection of PRMT1i ± PARPi, BETi, HDACi, EZH2i, PRMT5i for trials
Response Monitoring & Resistance Surveillance	Detect efficacy & emergent resistance	Serial plasma ctDNA, PBMCs, on-treatment biopsies	ctDNA molecular response, PRMT5 upregulation, splicing signatures, pathway bypass	Adjust therapy, identify resistance early, optimize dosing or combination strategy
Statistical & Biomarker Analysis	Ensure robust interpretation & validity	Pre-specified analysis plan	Continuous and categorical biomarker analysis, hierarchical testing	Supports validation of predictive and pharmacodynamic markers; prospective clinical validation remains limited
Regulatory & Safety Considerations	Facilitate companion diagnostic development & monitor toxicity	Clinical lab assays, safety labs	Hematopoietic function, liver toxicity, DNA damage markers, H4R3me2a pharmacodynamic monitoring	Ensures regulatory alignment, patient safety, reimbursement strategy
Post-Approval Surveillance/Real-World Evidence	Capture long-term safety, rare resistance patterns, and biomarker utility	Registries, RWE databases	Clinical outcomes, biomarker performance, adverse events	Refine exploratory biomarkers, evaluate long-term safety, and optimize patient selection

### Safety, toxicity, and translational challenges of PRMT1 inhibition

Despite encouraging preclinical activity, the clinical translation of PRMT1 inhibitors remains challenged by toxicity, selectivity, resistance, and the context-dependent biological functions of PRMT1. As PRMT1 is the predominant Type I arginine methyltransferase regulating transcription, RNA processing, DNA repair, immune signaling, and cellular metabolism, systemic inhibition may disrupt essential physiological pathways (1, 13). One major concern is hematologic and genomic toxicity. PRMT1 contributes to hematopoietic homeostasis and DNA damage repair through methylation of substrates such as BRCA1, MRE11, and 53BP1. Therefore, prolonged inhibition may impair erythropoiesis, immune cell function, and genomic stability in normal tissues (48–50, 125). Another challenge is limited inhibitor selectivity. Due to the structural similarity among PRMT family members, many compounds exhibit overlapping activity against multiple Type I PRMTs. For example, pan-Type I inhibitors such as MS023 suppress PRMT1, PRMT3, PRMT4, and PRMT6 simultaneously, potentially causing broader epigenetic dysregulation and off-target toxicity (111). In addition, indirect perturbation of PRMT5-associated pathways may disrupt RNA splicing and transcriptional fidelity (93). Adaptive resistance mechanisms further complicate therapeutic efficacy. Compensatory upregulation of PRMT5 or alternative methyltransferases may restore oncogenic methylation signaling following PRMT1 inhibition. Likewise, alterations in S-adenosyl methionine (SAM) metabolism and epigenetic rewiring may reduce inhibitor sensitivity and promote acquired resistance (93). Importantly, PRMT1 also exhibits context-dependent tumor-suppressive functions through regulation of oxidative stress, apoptosis, necroptosis, and mitochondrial homeostasis in selected cancer settings (70, 72, 73). Consequently, indiscriminate PRMT1 inhibition may inadvertently suppress protective cellular pathways in certain tissues or tumor subtypes. Collectively, these findings highlight the need for biomarker-guided and context-specific therapeutic strategies. Approaches such as isoform-selective inhibitors, intermittent dosing, rational combination therapies, and pharmacodynamic monitoring may improve therapeutic windows while minimizing systemic toxicity and resistance.

## Emerging technologies

Technological advancements have revolutionized our understanding of PRMT1 role in cancer regulation, providing molecular insights for targeted drug delivery approaches. High resolution Cryo-electron microscopy (cryo-EM) and X-ray crystallography have offered total structural details of PRMT1, revealing critical structural components like the double E circle and THW motif essential for substrate recognition and enzyme activity. These perceptivities have driven the development of specific inhibitors, such as MS023 and GSK3368715, targeting PRMT1’s SAM- binding site with higher specificity (119, 126). Fragment based drug discovery has further identified allosteric interaction sites, enabling modulation of PRMT1 activity while minimizing off-target activity and enhancing bioavailability. Whereas, epigenomic profiling techniques, including CUT&RUN and single-cell RNA sequencing (scRNA-seq), have impacted PRMT1 research by allowing precise mapping of histone modifications like H4R3me2a and delineating PRMT1 role in cancer heterogeneity. These approaches expounded cell specific functions, particularly in regulating cancer stem cells and immune regulation, and its role in cancer microenvironment (127, 128). Moreover, single cell technologies have enriched PRMT1 role in transcriptional regulation and chromatin accessibility, advancing our understanding on its functions in cancer regulation and drug resistance. CRISPR based tools have further illustrated PRMT1 role in cancer. Genome-wide knockout screens have linked PRMT1 as a synthetic lethal target in cancers driven by EZH2 and BRD4 mutations (129). CRISPR interference (CRISPRi) and epigenome editing using dCas9-PRMT1 complex have provided mechanistic insights into PRMT1 mediated arginine methylation, enhancing our understanding of its transcriptional regulation in oncogenesis (130). Similarly, proteomics has identified various new PRMT1 substrates, revealing its influence on multiple cellular pathways, including transcription, RNA metabolism, and DNA repair. Quantitative mass spectrometry approaches, such as SILAC and TMT, have linked PRMT1 substrates ranging from histones to transcription factors and DNA repair enzymes. Proximity labeling methods like BioID have elucidated PRMT1 interactome, uncovering its association with chromatin remodelers like SWI/SNF to regulate transcription and RNA processing (101, 131). Interestingly, artificial intelligence (AI) and multi-omics integration is arising as transformative strategies in PRMT1 research. AI driven models have mapped new substrates and regulatory pathways, expediting the discovery of synergistic therapeutic regimes and inhibitor designs. Spatial multi-omics technologies, integrating transcriptomics, epigenomics, and proteomics, have revealed PRMT1 role within cancer-immune network and stromal regulation, providing better insights into its role in cancer microenvironments (132, 133). Furthermore, real- time monitoring of PRMT1 role has been achieved using advanced live cell imaging, including FRET based biosensors and super-resolution microscopy, which have elucidated its dynamic interaction with chromatin during cellular processes such as transcriptional activation and DNA repair. Wherein, organoid and patient derived xenograft models, combined with CRISPR editing, have served as physiologically applicable techniques to estimate the therapeutic efficacy of PRMT1 inhibitors, assisting translational to clinical studies (9, 99, 134).

## Predictive AI–integrated future perspectives

Rapid advances in artificial intelligence (AI) and multi-omics integration are poised to transform how PRMT1 biology is understood and therapeutically exploited. Machine learning models trained on large-scale methylomics, chromatin accessibility (ATAC-seq), and single-cell transcriptomic datasets can now infer PRMT1 dependent regulatory states, predict substrate specificity, and identify context-dependent vulnerabilities that are not detectable through conventional analyses (135). Deep neural networks capable of modeling combinatorial chromatin features may help distinguish PRMT1 driven enhancer activation from secondary methylation events, enabling computational stratification of PRMT1 high tumors that are most likely to respond to PRMT1 inhibitors or PRMT1 targeted combination strategies. In parallel, AI assisted protein ligand simulations, such as AlphaFold2-driven pocket prediction and reinforcement-learning based molecular design, offer unprecedented opportunities to accelerate the development of next-generation PRMT1 inhibitors with improved selectivity and pharmacodynamic stability (135). Integrating PRMT1 regulatory signatures with multiomic clinical datasets through graph neural networks may further uncover cooperative dependencies with BET, DNMT, EZH2, and PRMT5 pathways, providing a computational roadmap for rational, patient-specific combination therapies. Additionally, AI-enabled digital pathology can quantify PRMT1 expression, H4R3me2a levels, and epigenetic remodeling patterns directly from histological slides, creating scalable biomarkers for PRMT1 inhibitor response prediction. Together, these emerging approaches position AI as a central engine for decoding PRMT1 function, predicting resistance trajectories, guiding drug discovery, and enabling precision oncology frameworks that personalize PRMT1-targeted epigenetic therapy.

## Conclusions

PRMT1 has emerged as a major epigenetic regulator with dual and context dependent role in cancer. Its capability to mark asymmetric arginine methylation impacts a wide array of molecular processes, including chromatin remodeling, transcriptional regulation, RNA splicing, and DNA damage repair. In numerous cancers, PRMT1 acts as an oncogene by promoting chromatin availability and driving the expression of pro-oncogenes. Nevertheless, in specific microenvironment, it can exhibit anti-cancer activity, emphasizing its dynamic role across different cellular and tissue levels. The potential of PRMT1 as therapeutic target lies in crucial role as a modulator of cancer-specific transcriptional activity and in regulating tumor microenvironment. Advances in the development of specific PRMT1 inhibitors, such as MS023 and GSK3368715, mark a critical step toward clinical translation. Though, preclinical and early phase clinical studies have stressed, the duality in PRMT1 functions and the complexity of its crosstalk with other epigenetic regulators emphasize the importance of designing cancer-specific therapeutic strategies. Biomarker driven approaches will be essential to stratify cases and ensure that PRMT1 targeted therapeutics achieve maximum efficacy while minimizing adverse effects. Furthermore, PRMT1 interplay with signaling pathways and its influence on immune escape mechanisms present promising avenues for combination curatives. Integrating PRMT1 inhibition with immune checkpoint blocker or chemotherapy has the potential of enhancing therapeutic efficacy. These strategies must be coupled with comprehensive mechanistic studies to uncover resistance mechanisms and identify predictive biomarkers. Moving forward, the integration of cutting-edge technologies will advance our understanding of PRMT1 context-specific duality in cancer. By bridging basic mechanistic studies with translational research, PRMT1 has the potential to pave the way for precision oncology, offering new frontiers in cancer management.
